# ‘They don’t squeal, ‘Disabled’.’: using qualitative interviews to explore user perceptions of ‘stylish’ grab rails intended to promote healthy ageing in place in England

**DOI:** 10.1136/bmjopen-2024-093698

**Published:** 2025-03-25

**Authors:** Sarah Dickson, Kate Gibson, Mitra Cheraghi, Andrew Kingston, Katie Brittain

**Affiliations:** 1 Population Health Sciences Institute, Newcastle University, Newcastle upon Tyne, UK

**Keywords:** Aging, Aged, Frail Elderly, Health Services for the Aged, QUALITATIVE RESEARCH

## Abstract

**Abstract:**

**Objectives:**

This qualitative study seeks to answer the question: How do older adults use and perceive home adaptations, specifically grab rails designed to blend into the home environment and avoid overt associations with disability? The grab rails were provided by a large energy company. They were specifically designed to be discreet, stylish and have a dual purpose, with the aim of supporting healthy ageing through ageing in place.

**Design:**

A qualitative study using semistructured interviews, with thematic analysis.

**Setting:**

Interviews were conducted predominantly via telephone calls, between 5 June 2023 and 14 August 2023.

**Participants:**

33 participants took part in the study with a mean age of 64.2 years. Participants resided in the following regions across the UK: North East, North West, Yorkshire, East and West Midlands. Purposive sampling allowed diversity in gender, ethnicity and home tenure. The grab rails were installed at least 3 months prior to recruitment.

**Results:**

Many participants reported that using the grab rails helped their independence and safety in daily life. The aesthetics of the grab rails were positively appraised, specifically because they had a notably different outward appearance to standard grab rails. Participants were aware that declining mobility can be stigmatising, and they felt the grab rails mitigated this by being discreet and enabling them to present a home which they felt would be accepted by wider society. Participants felt this protected their identity, as they wanted to appear independent to wider society. However, some participants were unaware that the grab rails had been designed with a duality of purpose or were apprehensive towards the functionality of these grab rails. This apprehension may have stemmed from the home adaptations being provided by a large energy provider, or because the discreet design of the grab rails made their intended purpose less overt.

**Conclusions:**

While home adaptations with a discreet and stylish aesthetic are valued by older people, our findings highlight that there are issues with commodifying home adaptations. Furthermore, we demonstrate the importance of addressing the social stigma associated with ageing-related home adaptations.

**STRENGTHS AND LIMITATIONS OF THIS STUDY**

- This study was unique in its exploration of grab rails, specifically designed to be discreet and ‘stylish’, and provided by a private sector energy company.
- The use of a purposive sample allowed for diversity among participant characteristics.
- The study only considered the views of those living in selected parts of the UK.
- Declining mobility is an issue most typically experienced by those who are very old. The participant mean sample age for this study was relatively young (64.2 years).

## Introduction

 Home adaptations support individuals to age in place, with increased independence and safety^1^ and can facilitate healthy ageing. Ageing in place is where individuals remain at home instead of an institutionalised setting. The opportunity to do so should be regardless of age, financial status or ability.^2^ Promoting healthy ageing and ageing in place is a critical goal on international policy agendas,^3^ and most notably driven forward via the UN Decade of Healthy Ageing.^4^

However, the attitudes of older individuals towards home adaptations are nuanced and often contradictory. Some older people view home adaptations positively, considering them a means to preserve or restore their independence.^5^ Conversely, the often-clinical outward appearance of home adaptations is considered unappealing.^6^ Negative attitudes towards ageing^7 8^ have permeated into views towards home adaptations, especially those aimed at older people. Some older people view home adaptations as a symbol of frailty^9^ and a visual reminder of ageing and vulnerability.^5^

Alongside these conflicting views surrounding home adaptations, there are questions as to who the most appropriate provider is. The UK’s current process for home adaptations is complex and varies regionally.^10^ Long wait times and the cost of home adaptations can make home adaptations inaccessible.^1 5^ This will continue to be a challenge, particularly as the number of people aged 75 or older residing at home without adaptations despite requiring them has risen from 36% in 2014–2015 to 51% in 2019–2020.^1^ Given the UK’s ageing population,^11^ the unmet and increasing demand for home adaptations is increasing; this is an urgent issue.

In light of these issues, alternative providers, such as the private sector, have been called on to ease the burden on the UK’s health and social care services. Indeed, recent investments have centred on supporting businesses to develop and deliver market innovations to maximise commercial opportunities. This forms part of commitments to the UK government’s Ageing Society Grand Challenge to enable people to enjoy an extra 5 years of healthy, independent living by 2035.^12^ Homes for Living (HfL) is one of five trailblazer projects involved in the UK Research and Innovation (UKRI) Challenge fund.^13^ One of the aims of the UKRI Challenge fund is to address the societal challenge of an ageing society. The HfL scheme aims to promote healthy and independent living at home^13 14^ and was facilitated by a large energy company.

Private sector involvement could be considered in the public interest, as not being solely reliant on one sector could positively impact cost, quality and effectiveness.^15^ However, the involvement of the private sector within the health and social care arena raises concerns about the ‘commodification of care’,^16 17^ particularly to vulnerable groups.^18^

## Methods

This paper presents interview data from a qualitative substudy as part of a wider quantitative study, the HfL project, which was funded by UKRI.^13^ The study assessed the impact of the HfL scheme, which provided home adaptations to eligible individuals and was facilitated by a large energy company. All home adaptations supplied were specifically designed to be covertly used as grab rails but also have a dual purpose. Eligibility to participate in the HfL scheme included being over 50 years old, on means-tested benefits, and living in the North East, North West, Yorkshire, East or West Midlands of the UK.^14^

This qualitative study seeks to answer the question: How do older adults use and perceive home adaptations, specifically grab rails designed to blend into the home environment and avoid overt associations with disability? This paper specifically focuses on the experiences of 33 participants who were supplied by HfL with one or more of the following products: The Mirror, External Rail, Corner Shelf, Toilet Roll Holder.^19^ These grab rails were designed to be ‘discreet, dual-purpose and stylish’ and used as everyday household items.^19^ In addition to the grab rail function, the mirror had a reflective surface and a shelf for storing toiletries, the external rail could hold a potted plant, the corner shelf held toiletries in the shower and the toilet roll holder could be used like a standard toilet roll holder.

Participants were purposively sampled to ensure representation across categories such as ethnicity, gender and housing tenure. To enable us to fully understand their impact over time, in addition to the eligibility criteria outlined above, eligible participants were required to have products installed in their homes for a minimum of 3 months prior to recruitment.

Semistructured telephone interviews were conducted with all participants, bar one who requested a video interview. Interviews were conducted by MC and SD between 5 June 2023 and 14 August 2023. They lasted between 19 and 60 min, with an average duration of 33 min. Interviews explored participants’ perceptions of their daily life, their home, prior home adaptations, their experiences of the HfL grab rails and the overall scheme. The full interview guide is available as an online supplemental file. With participant consent, interviews were audio recorded and transcribed verbatim. Following this, SD and MC anonymised and checked the accuracy of all transcripts. All individuals involved in the wider HfL scheme received specified home adaptations free of charge in exchange for their participation. Participation in the qualitative substudy was voluntary, and following their interview, participants were reimbursed with a £50 shopping voucher.

Data collection and analysis occurred concurrently. Following each interview, MC and SD documented their post interview reflections. These reflections captured any initial thoughts to be of salience. A thematic approach to analysis was undertaken. This was a three-stage process; first the data were coded, then descriptive themes were developed, and finally analytical themes were generated.^20^ NVivo V.14 was used to code the data and themes were developed from this. KB, KG, MC and SD regularly met to discuss emerging themes and develop the final analysis. This iterative, multistage process and continued discussion helped ensure rigour and validity of data interpretation.

### Patient and public involvement

Public involvement was achieved with the assistance of Voice, an organisation in the North East of England, set up to involve members of the public in research and policy-making processes.^21^ Four workshops were conducted with Voice members who provided feedback on the project, findings and dissemination material. Their involvement with the dissemination material improved its suitability for a lay audience. Subsequently, amendments were made to the language and content of the final leaflet.

## Results

Participant characteristics are presented in table 1. The gender distribution was relatively balanced with a slight male majority: 55% male (18 participants) and 45% female (n=15). Age-wise, most of the participants were between 50 and 69 years old, accounting for 72% of the total, with 33% in the 50–59 age bracket and 39% in the 60–69 bracket. The ethnic composition was predominantly White, comprising 76% of the participants (n=25), followed by Asian or Asian British at 18% (n=6). Other ethnic groups represented were black/African/Caribbean/black British and other ethnic group, each making up 3% of the sample.

**Table 1:** Participant characteristics

	Participants (n=)	Percentage (%)
Gender		
Male	18	55
Female	15	45
Age		
50–59	11	33
60–69	13	39
70–79	5	16
80–89	4	12
Ethnicity		
Asian or Asian British	6	18
Black/African/Caribbean/Black British	1	3
Mixed/multiple ethnic groups	0	0
White	25	76
Other ethnic group	1	3
Home tenure		
Homeowner	18	55
Renting—social housing	7	21
Renting—local authority	5	15
Renting—private rent	3	9
Home occupancy		
Lives alone	12	36
2-person household	15	46
3-person household	4	12
4-person household	2	6
Index of Multiple Deprivation (IMD) Quintile*		
Most deprived	12	36
Highly deprived	7	23
Moderately deprived	5	16
Less deprived	7	19
Least deprived	2	6
Total	33	

IMD is the official measure of relative deprivation for small areas in England and takes multiple factors into consideration, such as employment, health and crime. On the scale, 1 is most deprived and 5 is least.^34^

* Index of Multiple Deprivation (IMD) was included as a characteristic, as individual barriers to healthy ageing can include poor health, social and financial problems.^35^

Regarding home tenure, 55% of the participants were homeowners (n=18), while the rest were renters, split among social housing (21%), local authority housing (15%) and private renting (9%). Home occupancy data revealed that 36% of participants lived alone, 46% resided in 2-person households and smaller percentages lived in larger households.

From a socioeconomic perspective, based on the Index of Multiple Deprivation, a considerable segment of our study population belonged to deprived areas: 36% from the most deprived quintile and 23% from the highly deprived quintile. This highlights the potential influence of socioeconomic factors on the research outcomes, given that nearly 59% of the participants came from the two lowest quintiles of socioeconomic status.

Overall, most of the sample had health issues and poor mobility. Health conditions included arthritis, depression, epilepsy and fibromyalgia. Most participants reported having other housing adaptations or aids in their home. Prior home aids included grab rails, perching stools and wheelchairs. Housing modifications often cited were wet rooms, installation of a downstairs toilet and stairlifts.

Findings are grouped into the following themes:

1. Grab rails and daily life.
2. Product aesthetics and distance from ‘disabled products’.
3. Concerns towards the grab rails and HfL scheme.

These themes are presented below alongside supporting participant quotes, which are contextualised with demographic information.

### Theme 1: Grab rails and daily life

Many participants felt the HfL grab rails made a positive difference to their everyday life. ‘Safe’ and ‘trust’ were words frequently used when discussing how they felt using these grab rails, particularly the corner shelf and external rail. In turn, this increased their confidence to undertake daily activities, such as washing and leaving the home.

[Corner shelf] ‘It’s lovely. I can wash with one hand and hold the rail with the other. So, I’m safe now.’ P15, Male, 80yrs

[External rail/corner shelf/toilet roll holder] ‘…I feel that I can trust them.’ P34, Female, 56yrs

Participant evaluations of the external rail highlighted how using the product made mobility ‘easier’, and as a result, allowed them to leave their homes. One participant described the product as a ‘helping hand’ and another spoke about the increased independence the product provided.

[External rail] ‘Well yeah, obviously this product makes it a bit easier for me to get around. It’s easier for me to get out the house.’ P25, Male, 70yrs

[External rail] ‘If I go out in the garden or whatever, it’s a helping hand again, you know? I found that when I go and do the bins or whatever, that also helps.’ P4, Female, 60yrs

[External rail] ‘It gives me an independent life. I couldn’t go into the garden without that handrail.’ P6, Female, 87yrs

Some participants directly aligned the HfL grab rails with enabling them to continue living at home independently. This, in turn, engendered a realisation that ageing in place could be facilitated with home adaptations.

[External rail/corner shelf] ‘Yes, it’s made us feel more secure that we’re able to stay here for longer, yes. The products have really made a difference, yes.’ P24, Female, 58yrs

‘…it changed my life. It gives me an independent life. If we wouldn’t have had all these products, I could not live that independently.’ P6, Female, 87yrs

[toilet roll holder/external rail] ‘It’s made me realise that there are always options and avenues you can explore. You’re not stuck with the house; you can alter and amend the house to fit your condition.’ P28, Male, 76yrs

Most participants felt safe using the HfL grab rails, which in turn allowed them to be more mobile and complete daily activities with increased independence. Some participants positioned the grab rails as facilitating their ability to age in place.

### Theme 2: Product aesthetics and distance from ‘disabled products’

Most participants positively appraised the aesthetics of the grab rails and, in line with the sentiment behind their design, considered them ‘stylish’ and ‘trendy.’

[Corner shelf/toilet roll holder] ‘But the shelf and the grab rails incorporating the toilet roll, they look so stylish. Even though I’m 82, I’m not very old-fashioned, if you know what I mean.’ P5, Female, 82yrs

[Corner shelf] ‘It looks like something very trendy, you know? It looks really good. I like that.’ P23, Male, 53yrs

A strong, recurrent theme across the sample was that participants felt that the ‘modern’ outward appearance of HfL grab rails meant they did not look like ‘standard’ disability equipment.

[External rail/mirror] ‘But these are quite modern and don’t stand out as a mobility helping aid, if you will.’ P29, Female, 50yrs

[mirror rail] ‘The towel rail, it doesn’t even look like it’s disability equipment, it just looks like a towel rail.’ P3, Female, 56yrs

[External rail/corner shelf/toilet roll holder] ‘I think the fittings in our house go very well with the house. And they look fine. They don’t look…don’t tell, at the first going in, there is somebody who can’t walk so well.’ P6, Female, 87yrs

[Corner shelf/toilet roll holder] ‘They don’t squeal, “Disabled.” P5, Female, 82yrs

Because the grab rails do not ‘squeal disabled’, part of their appeal lay in their capacity to blend in with participants’ domestic environments. Indeed, one participant explicitly highlighted that the grab rails prevented her home from resembling a hospital, compared with ‘standard’ home adaptations. Thus, they were valued for their capacity to avoid blurring the line between home and hospital.

[External rail/corner shelf/toilet roll holder] [In relation to products] ‘I then don’t have to turn my home, as I get older, into a hospital…and not have to limit myself to the sort of white, plastic-coated, which I detest.’ P34, Female, 56yrs

Significantly, this recurrent preference for distance from ‘standard’ disability equipment positively impacted participants’ sense of well-being:

‘We feel more included with well-designed products, as opposed to somebody going, “Oh, you need a handrail?” and they put a bit of scaffolding pole in that’s ugly…’ P13, Male, 63yrs

[External rail/corner shelf/toilet roll holder] ‘But if these were around when I was 17 or 18, when I started becoming unwell, it would have made so much difference to the way I felt about myself and my home, and the embarrassment that I have surrounding my disability.’ P34, Female, 56yrs

[toilet roll holder/external rail] ‘And, oddly enough, we had a few friends round the other night and I thought they’d mention the rails. You know, “Come on, you old devil,” but nothing. No, they said, “Well, we didn’t even notice them.” So, you know, I feel a bit better about that.’ P28, Male, 76yrs

Notably, the participant above focuses on the subtlety of the HfL products; he ‘felt better’ that visitors to his home had not noticed the grab rails. Other participants echoed this sentiment:

[Corner shelf/ external rail] ‘I think that’s marvellous, because, you know what I mean? You don’t have to tell people that they’re disability aids, you can have people in your home, and you know what I mean, they wouldn’t know unless you told them, yes.’ P24, Female, 58yrs

Most participants positively appraised the aesthetics of the grab rails. The HfL grab rails were considered stylish, and as such, distinctly different from standard home adaptations. A preference towards a divergence from adaptations was due to them embedding better within the rest of their homes. A significant aspect of their appeal was that the discreet design of the grab rails allowed for declining mobility to be hidden both to visitors to the home and, for the external rail, wider society.

### Theme 3: Concerns towards the HfL grab rails and wider scheme

Involvement in the installation process was a key mechanism in shaping participants’ confidence in using the grab rails. Yet, participants had mixed experiences. Some participants were consulted during the grab rail installation.

‘They asked me which height I would like it. I sat in the chair and put my hand where and I said, “There”, so he put it there.’ P31, Female, 67yrs

However, others were not consulted. For example, P20, a wheelchair user, was not consulted in the installation process, which negatively impacted the usability of the grab rail. The external rail was installed at the wrong height and resulted in him becoming injured.

‘The one at the front door could do with taking down…I’ve smashed my head on it a few times.’ P20, Male, 54yrs

P18 shared similar frustrations about not being involved in the installation process:

‘What they could have done was ask me where I wanted it or—If I sit there on the loo, then I could have been able to judge where I wanted it, but they didn’t ask me.’ P18, Male, 63yrs

HfL grab rails were specifically designed to be dual purpose. Their ‘discreet’ design hid their grab rail function, allowing them to masquerade as a standard household fixture. However, across the product range, multiple participants were unaware of the duality of purpose. This unawareness was shown for both the primary and secondary function.

[mirror rail/toilet roll holder after they had been fitted] ‘To be honest, I never looked and saw them as adaptations or handles until somebody else pointed it out.’ P20, Male, 54yrs

[External rail] ‘I mean we’ve got plant pots all over the house and outside, my wife loves plants, but we didn’t realise that.’ P39, Male, 71yrs

However, other participants were aware of the grab rail function, but displayed apprehension nevertheless. This was particularly in relation to whether the products could support their weight.

[toilet roll holder] ‘…I don’t want to lean on it too much, in case it breaks…I wouldn’t feel safe holding on to just that…’ P22, Female, 71yrs

[Corner shelf] ‘No, the rail’s not strong enough to support me.’ P26, Male, 67yrs

P25 explained his apprehension towards displaying a plant on his external rail, as this could obstruct the primary function of being used as a grab rail.

‘…but you know if you put a plant in it, that’s going to grow and it’s going to make it difficult to grab hold of it, without damaging the plant.’ P25, Male, 70yrs

Not only was there apprehension towards the grab rails, but some participants were also apprehensive towards the HfL scheme. Despite a widespread sense of positivity and gratefulness towards the HfL scheme, prior to installation, some participants were concerned the HfL scheme was not a genuine opportunity.

‘The big worry was, like most scams, it seemed too good to be true…’ P12, Male, 60yrs

‘Again, because of the way the world is, unfortunately, I have told to me all the time about people with disabilities being on the end of cons, tricksters…And my only alarm when I first came across the scheme, I thought, “This just seems too good to be true.”’ P13, Male, 63yrs

## Discussion

### Statement of the principal findings

This study sought to describe and explore older people’s perceptions of grab rails, which aimed to promote healthy ageing. Most participants considered that the HfL grab rails positively impacted their daily lives, with increased feelings of safety, ease and independence. Grab rails are part of a range of home adaptations that are used to support ageing in place. Our findings echo prior literature which found home adaptations can support healthy ageing, helping with falls prevention and improving older persons’ safety within their home.^22^ This can lead to positive feelings in improved quality of life and reduced fear of falling.^23^

Another prior study, which provided home adaptations alongside occupational and physical therapy sessions, was found to improve health and functional abilities in older people.^24^ While our study did not include therapy sessions, it displayed similar findings in revealing that grab rails improved participants’ perceptions of safety, independence and that the ‘stylish’ design had a positive mental impact.

Aside from the broader goal of improved perceptions of safety, participants highly valued the aesthetics of the grab rails, which consequently positively impacted some individuals’ well-being. Most prominently, participants favoured that these grab rails were designed to not look like standard ‘disabled’ adaptations. This was considered desirable, as the ‘stylish’, ‘discreet’ and ‘modern’ appearance of the HfL grab rails allowed participants to conceal their declining mobility. It promoted feelings of inclusion and prevented reminders of previous hospital admissions. This echoes research exploring the use of medical equipment in the home which found that clinical aesthetics symbolised the medical world. In turn, this intruded on personal environments and family life.^25^ This blurring of domestic and clinical boundaries, together with a stigma towards home adaptations, could present a challenge to healthy ageing and ageing in place. Furthermore, our data revealed additional challenges of discreet home adaptations, including participants’ lack of awareness about the products’ dual functions or their hesitancy to use them.

### Strengths and weaknesses of the study

This study engaged directly with older people who had used the HfL grab rails, which meant rich, qualitative data was obtained. However, researching ageing presents methodological challenges^26^ as older people are less likely to express concerns about unsuitable housing.^1^ Furthermore, the insight obtained was limited to topics which participants recognised and felt comfortable sharing.^27^ Older people can underreport home difficulties due to fears of seeming incapable or the risk of being moved into residential care.^27^ This is relevant to this study, as many HfL grab rails related to personal tasks, such as toileting and bathing, and were used to help reduce the risk of falls in the home.

Furthermore, older people may feel uncomfortable voicing their concerns, fearing it may seem as though they are complaining and ungrateful.^26^ To address this issue, interviewers clarified they were independent of the provider and assured participants that their home adaptations would remain free, regardless of the feedback they provided. Finally, it is important to note the mean age for participants who formed the qualitative grab rail data was 64.2 years. This is relatively young to be classified within the older person category.

### Strengths and weaknesses in relation to other studies

The stigma attached to ageing and the use of home adaptations is well established in the literature.^6 28 29^ Levy and Banaji, for instance, note that stigma and ageing co-occur and negative associations, such as vulnerability, go largely unnoticed and continue to exist in society.^30^ These grab rails could, therefore, enable older people to manage this stigma, by both helping them maintain their independence and avoid being seen as vulnerable. Participants in our study favoured more discreet grab rails. By emphasising a preference for designs which do not ‘squeal disabled’, they alluded to an awareness of this stigma. Participants wanted friends visiting their home not to notice their grab rails, and one of our participants ‘felt better’ that no one had mentioned them. Moreover, participants were all too aware of the consequences of one’s declining mobility being ‘seen’. It also impacted on their perceived physical safety, with concerns that an external rail ‘advertised’ that a disabled person lived at the property. This concern is also apparent in the literature.^31^ This forms part of the wider view that home adaptations symbolise frailty,^9^ vulnerability^5^ and physical decline.^28^ Thus, the discreet design of HfL grab rails supports more than aesthetics; it enables users to maintain an identity of independence in their living environment.

### Possible explanations and implications for clinicians and policy-makers

Our study supports policy recommendations, which emphasise the need for integrated, well-considered and supported home adaptations to facilitate ageing in place.^32^ These grab rails were purposefully designed to be discreet and have a duality in purpose. However, our data showed issues surrounding participants being unaware of the duality of purpose or apprehension to use them. This raises questions about the function of grab rails with an overtly ‘disabled’ appearance. That is, traditional grab rails that are ‘white, plastic coated’ and ‘squeal disabled’ may have an important function of making their intended purpose very overt, so individuals instinctively know how to use them.

Equally, this apprehension towards using products could be the result of a wariness towards the HfL scheme or lack of confidence in the processes by which the grab rails were installed. This raises a potential consideration if home adaptations are to be supplied by private sector companies. The private sector ethos of efficiency and profitability may not be suitable for application to a grab rail provider. Our data demonstrated the importance of consultation in the installation process. Those who were given choice utilised the grab rails better than those who were given no choice, which for P20 resulted in injury. Markets are based on competition and responding to financial incentives, which is in tension with the ethos of social care.

Age UK highlights that older people are wary towards individuals or companies offering goods or services.^33^ As private sector companies increasingly become alternative providers, attention must be paid to developing trust and better aligning with delivering care. A more personalised approach could have allayed some participants’ fears. A one-size-fits-all approach cannot attend to the diverse needs and understandings of those ageing in place.

### Unanswered questions and future research

Literature suggests that renters and homeowners are faced with different challenges for accessing home adaptations.^1 23^ While there was diversity of home tenure in our sample, participants did not discuss topics such as landlord approval or concerns over resale value. This could be explored in future research, especially in relation to grab rails designed to be dual purpose and discrete.

The positive impact the HfL grab rails had on some participants’ daily life, often related to independence or mobility. This was apparent despite the relatively young mean sample age of 64.2 years. Future research could ensure the purposive sampling considered age as a factor, so that the data could focus on those in later old age.

## Conclusions

Overall, these grab rails supported participants to age in place by affording them with the confidence to move with safety, ease and independence, both inside and outside of the home. Participants positively appraised the grab rails for their capacity to blend into their domestic environments. They were valued because they did not serve as a visual reminder of actual or impending loss of mobility to participants themselves, their immediate social networks and wider society. However, while the discreet design of the grab rails enabled participants to present a home (and identity) of independence, participants alluded to an awareness of the stigma associated with ageing and decline. As such, our findings highlight a need to address the stigma associated with ageing and declining mobility at a societal level. While the visual appeal of the products allowed participants to present a healthy ageing identity, an aesthetic design which centres on concealing declining functionality perpetuates the stigma attached to functional decline rather than addresses it.

## supplementary material

10.1136/bmjopen-2024-093698 online supplemental file 1

## Data Availability

Data are available on reasonable request.
