# Supplementary material for: ‘They don’t squeal, ‘Disabled’.’: using qualitative interviews to explore user perceptions of ‘stylish’ grab rails intended to promote healthy ageing in place in England
Source: BMJ Open. 2025 Mar 25;15(3):e093698. doi: 10.1136/bmjopen-2024-093698 (PMC11938234; doi:10.1136/bmjopen-2024-093698)
Supplement: online supplemental file 1 [file bmjopen-15-3-s001.docx]

## Introduction

- Hi, is this [insert participant’s name]?
- Hi, it’s [insert researcher’s name]. I’m a researcher from Newcastle University. We arranged to have a chat about [large energy supplier name removed] Homes for Living study and your experiences of the products you had installed. Are you still okay to do this?
- Great, thank you. Also, I just wanted to let you know that this phone call will be recorded but this will be done in accordance with Newcastle University’s ethical guidelines. Are you still okay to proceed?
- **Start telephone recording**
- Great. Following participant’s approval, I have started the phone call recording.
- **This is participant number. [………………….]**
- I will now read through the participant consent form and if you could please say yes or no to each of the statements.
- **Complete consent form and in box that asks for signature, write ‘verbal consent recorded.’**
- Everything you say is confidential. You can stop the interview at any time. If you don’t want to answer any questions, you don’t have to. Just let me know and we can skip that question and move onto the next one.
- **Also, to clarify, Newcastle University is separate and independent from [large energy supplier name removed], so please feel comfortable to openly discuss anything which you have been happy or unhappy with.**
- Before we begin, do you have any questions or concerns at all?
- Brilliant, we can now begin the interview.

## About you and your home

I’d like to start by asking you some questions about you and your home.

1. **Could you tell me a little bit about yourself?**

General health, household/family structure, things you enjoy (who you do these activities with, not just physical but mental activities, what sort of activities did you enjoy when you were younger), how long have you lived in the area, could you talk me through a typical week for you and your daily activities?

1. **Could you tell me about your home?**
2. How long you have lived in your current home?
3. Description of home (including access)
4. Do you currently have any challenges getting around your home?
5. How do you feel about your home? Is there anything you particularly like or dislike? Anything that you would change?
6. Is it important to you that you can stay in your home in the long-term? Why/why not?
7. Is there anything you would do to your home to help you stay in your home longer or live here more comfortably?
8. **Apart from these [large energy supplier name removed] products, do you have any other adaptations at home? Or has your home been modified at all to help you live here more comfortably?**

*If yes:*

1. What are they and why did you get them? (Could be a product or a modification e.g. doors widened)
2. How did you find out about them, where did you get them, who did you buy them from?
3. How do these products compare to other adaptations you have in your home, or that you know about?
4. What was the installation process like for these products? Did you get any choice on where/how they were fitted? Did you have a prior consultation before the install? (If it was a family member who fit them, ask more questions about that)

## The products

1. **Could you please tell me about what you’ve had installed from [large energy supplier name removed]? (open question – no prompts)**
2. **NB These sets of questions are to be asked for each product.**
3. Could you describe [the product] and where it is found in your home?
4. Do you use it? If no, why not? If yes, how? Did you use it straightaway? Do you use it for any other purposes? Does anyone else use it?
5. Did you have any worries before you got the product? Why/Why not?
6. Do you have any concerns about [the product] now?
7. Has it made a difference to your daily life? How? (E.g. are you able to do anything you couldn’t do before, or do things with less difficulty?)
8. How do you feel about the way it looks? Do they fit in well within your home interior?
9. Could the product be improved? Would you recommend it? Why/why not?
10. How does it compare to other products/adaptations you’ve had?
11. **Has the installation of these products impacted on the way you feel about living in your home?**
12. Have the products impacted on the way you feel about your home itself?
13. How about you as a person, has having these products changed the way you see yourself? If so, how/why?
14. What about in the future, how will these products fit into your life and home over the long term?
15. Would you ever think about getting home adaptations before you needed them, as a way of planning for the future?
16. **Could you tell me about how you came across H4L and these products?**
17. Did you register your interest with [large energy supplier name removed], or did someone contact you?
18. How did you feel about being contacted about these products? (Can you remember what happened when they contacted you and what sort of questions they asked?) OR why did you contact [large energy supplier name removed] about these products?
19. How did you decide which products to have or not to have?
20. Was there anything that you wanted that you couldn’t have?
21. What was the installation process like for these products? Did someone come out to see you and your home first? Did you get any choice on how these products were fitted?
22. How do you feel about an energy provider providing products designed to help people live independently at home?
23. **If the products weren’t free of charge, would you pay for them? Why/why not?**
24. If you would pay for it/them, would spending money on these types of products be a priority for you? How would they compare to other things you spend money on like bills or food or days out?
25. If answers with just yes/no, say ‘Do you mind if I ask why? Or could you tell me a little more about why this is?’

**9. Has the [large energy supplier name removed] Homes for Living project changed the way you think about getting older?**

**Feedback/Debrief**

- Is there any other information that you would like to add? Do you want to go back to any of these interview questions to talk about anything in more detail? (Is there anything we want to go back to?) What did you think about the questions that we asked you for this interview?
- What made you take part in this interview?
- **Thanks for your time**

1. You will be sent a Thank you card and a £50 voucher for taking part. (If non-immediate risk support is needed, write contact details – such as Age UK – in card.)
2. Would you like a copy of your consent form? Findings to be published in March 2024.
3. **Any immediate risk support, follow guidance on spreadsheet. Complete spreadsheet following every interview conducted.**
