# Supplementary material for: ‘They don’t squeal, ‘Disabled’.’: using qualitative interviews to explore user perceptions of ‘stylish’ grab rails intended to promote healthy ageing in place in England
Source: BMJ Open. 2025 Mar 25;15(3):e093698. doi: 10.1136/bmjopen-2024-093698 (PMC11938234; doi:10.1136/bmjopen-2024-093698)
Supplement: Supplementary file 2 [file bmjopen-2024-093698.reviewer_comments.pdf]

PEER REVIEW HISTORY

BMJ Open publishes all reviews undertaken for accepted manuscripts. Reviewers are asked to complete a checklist review form and are provided with free text boxes to elaborate on their assessment. These free text comments are reproduced below.

ARTICLE DETAILS

Title (Provisional)

'They don't squeal, "Disabled." Using qualitative interviews to explore user perceptions of 'stylish' grab rails intended to promote healthy ageing in place in England.

Authors

Dickson, Sarah; Gibson, Kate; Cheraghi, Mitra; Kingston, Andrew; Brittain, Katie

---

VERSION 1 - REVIEW

Reviewer 1

Name Fyffe, Ian

Affiliation Fraser Health Authority, Long Term Care and Assisted Living Research Unit

Date 07-Jan-2025

COI None

I assume that the research question is "This qualitative study evaluates how older people used and perceived several home adaptations provided by HfL." This should be reworded to something more obvious to the reader and stated in the form of a question.

I would like the interview guide to be included as an appendix for replicability.

In the results of the abstract I would change "or because the grab rail's

discreet design made their intended purpose less overt." to "because the discreet design of the grab rails made"

Reviewer 2

Name Amal , Chakrabourty

Affiliation University of Sydney CAR, Faculty of Medicine and Health

Date 29-Jan-2025

COI None

Thank you for the opportunity to review this important paper that aims to explore older participants' perceptions of grab rails provided by a large energy company with the aim of supporting healthy ageing through ageing in place. I commend the work by the authors in undertaking and reporting this important piece of work.

Upon reading the manuscript I felt that there are some issues that need to be rectified to strengthen the paper. Here are some of my comments:

(1) Lines 3-4 on page 2 of 15: Title - Suggest revising the title with a positive tone using part from another participant's quote. One example of the revised title could be – "It gives me an independent life". Exploring 'stylish' home adaptations to promote healthy ageing in place: a qualitative study.

(2) Line 43 on page 3 of 15: UKRI Challenge Fund - define UKRI in the first instance.

(3) Line 56 on page 3 of 15: Homes for Living (HfL) project – already defined on line 43 so only abbreviated HfL can be stated here.

(4) Table 1 and its corresponding text should be moved to the Results section.

VERSION 1 - AUTHOR RESPONSE

Reviewer: 1
Mr. Ian Fyffe, Fraser Health Authority
Comments to the Author:
I assume that the research question is "This qualitative study evaluates how older people used and perceived several home adaptations provided by HfL." This should be reworded to something more obvious to the reader and stated in the form of a question.

This text has now been amended to reflect this comment and we hope the research question is now clearer. The amended text has now been included in both the abstract and in the main body of text;

This qualitative study seeks to answer the question: How do older adults use and perceive home adaptations, specifically grab rails designed to blend into the home environment and avoid overt associations with disability?

I would like the interview guide to be included as an appendix for replicability.

The interview guide is now included as a supplementary file.

In the results of the abstract I would change "or because the grab rail's discreet design made their intended purpose less overt." to "because the discreet design of the grab rails made"

This amendment has now been made;

This apprehension may have stemmed from the home adaptations being provided by a large energy provider, or because the discreet design of the grab rails made their intended purpose less overt.

Reviewer: 2
Dr. Chakrabourty Amal , University of Sydney CAR

Upon reading the manuscript I felt that there are some issues that need to be rectified to strengthen the paper. Here are some of my comments:

(1) Lines 3-4 on page 2 of 15: Title - Suggest revising the title with a positive tone using part from another participant's quote. One example of the revised title could be – "It gives me an independent life". Exploring 'stylish' home adaptations to promote healthy ageing in place: a qualitative study.

Thank you for overall feedback on our paper. We appreciate your suggestion to revise the title. We too believe it is important to highlight the positive impact home adaptations can have for healthy ageing. However, the study's central finding was that participants valued these grab rails primarily for their ability to blend in and avoid associations with disability. For example, traditional grab rails were seen as visual reminders of hospital stays and participants shared concerns regarding potential stigma from wider society for using outwardly 'disabled' products. Participants evidently valued these specific grab rails for their capacity to conceal declining ageing and mobility. As such, we feel including the quote 'They don't squeal, "Disabled." within our title captures this nuanced theme.

(2) Line 43 on page 3 of 15: UKRI Challenge Fund - define UKRI in the first instance.

The full acronym is now included in-text with a definition;

Homes for Living (HfL) is one of five trailblazer projects involved in the UK Research and Innovation (UKRI) Challenge fund[13]. One of the aims of the UKRI Challenge fund is to address the societal challenge of an ageing society.

(3) Line 56 on page 3 of 15: Homes for Living (HfL) project – already defined on line 43 so only abbreviated HfL can be stated here.

This has now been removed and the sentence reads;

This paper presents interview data from a qualitative sub-study as part of a wider quantitative study, the HfL project, which was funded by the UKRI[13].

(4) Table 1 and its corresponding text should be moved to the Results section.
This table and corresponding text has now been moved.
